# An Asymptomatic Case of Type B Aortic Dissection Developing Type A Dissection During Interfacility Transfer: A Case Report

**DOI:** 10.1002/ccr3.73053

**Published:** 2026-06-28

**Authors:** Kayo Sugiyama, Hirotaka Watanuki, Masato Tochii, Katsuhiko Matsuyama

**Affiliations:** ^1^ Department of Cardiac Surgery Aichi Medical University Hospital Nagakute Aichi Japan

**Keywords:** asymptomatic aortic dissection, retrograde type a aortic dissection, total arch replacement with frozen elephant trunk

## Abstract

During the transfer from a hospital to a tertiary hospital, patients with acute aortic dissection can deteriorate due to aortic rupture or extension of the dissection. Particularly when type B aortic dissection progresses to type A dissection, it can lead to serious complications. This case report that a 50‐year‐old man who was transferred to our institute for thoracic endovascular aortic repair for complicated acute type B aortic dissection developed retrograde type A aortic dissection and was successfully treated with total arch replacement using a frozen elephant trunk. The patient experienced retrograde type A aortic dissection during transfer for treatment of complicated type B aortic dissection while no changes in hemodynamics or symptoms were observed. After arrival at our institution, open surgical repair with total arch replacement using the frozen elephant trunk technique successfully treated the entry closure in the proximal descending aorta. The intraoperative direct echography showed no intimal tears in the ascending aorta, so total arch replacement using a frozen elephant trunk was chosen. The patient survived without any evident complications and was cured with satisfactory outcomes at a 5‐year follow‐up. Aortic dissection can worsen even when a patient has no symptoms or hemodynamic changes. When transferring a case of aortic dissection, caution is required regarding progression of dissection. Total arch replacement using a frozen elephant trunk for retrograde aortic dissection is effective in resecting intimal tears.

## Key Clinical Message

Aortic dissection can worsen even when a patient has no symptoms or hemodynamic changes. It is particularly necessary to be aware of the possibility that type B aortic dissection may progress silently to type A aortic dissection without new symptoms or instability. Total arch replacement using a frozen elephant trunk for retrograde type A aortic dissection is effective in resecting intimal tears.

## Graphical abstract

Total arch replacement using a frozen elephant trunk for retrograde type A aortic dissection was effective in excising the intimal tear located in the proximal descending aorta.

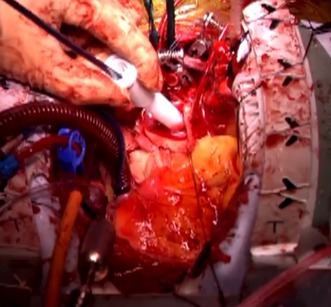

## 1 Introduction

Aortic dissection is a rare, life‐threatening aortic disease with an annual incidence of 2.5 to 15 cases per 100,000 people [1]. Acute type A aortic dissection (ATAAD) involves the ascending aorta, affecting cardiac tamponade and coronary perfusion, and requires emergency surgery. In contrast, acute type B aortic dissection (ATBAD) does not involve the ascending aorta and is primarily managed with conservative treatment. While ATAAD is characterized by severe pain radiating to the neck or jaw, stroke‐like symptoms, and severe chest pain, ATBAD is characterized primarily by severe pain in the back, abdomen, or between the shoulder blades [1].

Retrograde type A aortic dissection (RTAAD) involves the ascending aorta without an intimal tear and can be spontaneous or iatrogenic, resulting from open or endovascular aortic surgery [2]. Spontaneous RTAAD has been reported in 7%–25% of ATAAD cases [3], and patients with RTAAD according to reports do not present with the typical symptoms related to aortic dissection compared to antegrade type A aortic dissection [4]. Aortic wall fragility and an aortic diameter of 40 mm or greater are considered risk factors for RTAAD [2]. The 30‐day mortality rate of patients with RTAAD is 11%, which is significantly lower than that of patients with antegrade aortic dissection (34%) [5]. However, the absence of typical symptoms may lead to delayed detection and appropriate treatment.

The management of RTAAD is currently not standardized, with reports of successful treatment with optimized medical therapy, open surgery, and/or endovascular repair available in the literature [2]. To exclude the primary tear during the initial procedure, extensive aortic replacement is required (i.e., total arch replacement with frozen elephant trunk [TARFET] technique). Although TARFET is effective for repair of RTAAD, these procedures are still associated with high mortality and morbidity rates, particularly risks related to spinal cord injury [6, 7].

We encountered a case of asymptomatic RTAAD that was successfully treated with emergency TARFET.

## 2 Case Presentation

### 2.1 Case History

The patient was a 50‐year‐old man with untreated hypertension and a history of smoking. He had no history of heritable connective tissue disorders or family history of aortic disease. He was referred to a previous hospital with chest and back pain as a chief complaint. An electrocardiography revealed normal sinus rhythm without ischemic changes. A chest radiography revealed no cardiomegaly or pulmonary congestion but mediastinal enlargement. Although no heart murmurs or neurological deficits were detected, absent pulses were confirmed in both lower extremities. Upon arrival at the referring hospital, the patient's blood pressure was elevated at 180/110 mmHg, with a heart rate of 86 beats per minute and a respiratory rate of 18 breaths per minute.

### 2.2 Differential Diagnosis, Investigations and Treatment

A computed tomography (CT) revealed a dissection of the descending aorta (Figure 1a). No dissection was observed in the ascending aorta; its diameter was 42 mm, and although no organ malperfusion was noted, the true lumen was relatively small (Figure 1b). Consequently, the patient was transferred to our hospital to undergo thoracic endovascular aortic repair (TEVAR) for complicated type B aortic dissection. During the 50‐min transfer, systolic blood pressure was maintained between 90 and 100 mmHg. As symptoms of chest and back pain had resolved with antihypertensive medication, no worsening of symptoms was observed. Upon arrival at our hospital, the patient's vital signs were normal (blood pressure, 128/44 mmHg; heart rate, 80 beats per minute; body temperature, 36.4°C; and oxygen saturation, 96% on room air). Preoperative laboratory results at admission were as follows: hemoglobin, 13.8 g/dL; platelet count, 1.9 × 10^5^/μL; D‐dimer, 116 μg/mL; prothrombin time‐international normalized ratio, 1.2; activated partial thromboplastin time, 32 s; N‐terminal pro‐brain natriuretic peptide, 154 pg/mL; and C‐reactive protein, 0.85 mg/dL. However, after arrival at our hospital, a CT scan was performed to measure for TEVAR, revealing that the dissection had progressed to Stanford A (Figure 1c,d). Although cardiac tamponade, severe aortic valve regurgitation, or major organ malperfusion were not observed, the true lumen of the ascending aorta was relatively small; therefore, we decided to perform emergency open surgical repair. A primary tear in the aortic dissection was identified in the proximal descending aorta. Following a median sternotomy and the establishment of a cardiopulmonary bypass via the right femoral artery and vein, TARFET was performed. Upon opening the pericardium, no pericardial effusion was observed, and direct echography revealed no intimal tears in the ascending aorta, consistent with the CT images (Figure 2a). Upon opening the aorta, an intimal tear was identified in the proximal descending aorta, leading to the decision to perform TARFET for entry closure (Figure 2b). Postoperatively, the patient recovered without major complications and was discharged 2 weeks later. In this case, no antiplatelet or anticoagulant agents were administered.

**FIGURE 1:**
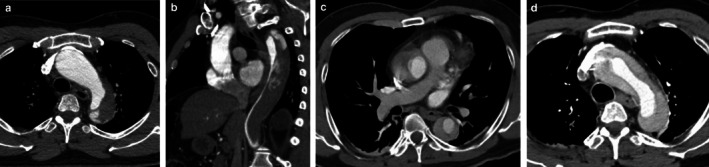
(a) Preoperative computed tomography before admission showing type B aortic dissection with no signs of aortic dissection in the ascending aorta. (b) Preoperative computed tomography before admission showing complicated type B aortic dissection with narrow true lumen. (c) Preoperative computed tomography after admission showing type A aortic dissection while it shows no signs of intimal tear in the ascending aorta. (d) Preoperative computed tomography after admission showing type A aortic dissection and not showing intimal tear in the ascending aorta and the aortic arch.

**FIGURE 2:**
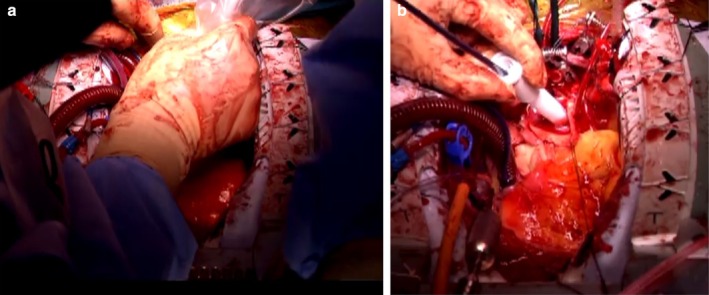
(a) Intraoperative direct echography showing no intimal tear in the ascending aorta. (b) Intraoperative view showing the insertion of a frozen elephant trunk for entry resection into the proximal descending aorta.

### 2.3 Outcome and Follow‐Up

Postoperative CT scans revealed no major complications, and the intimal tear was treated using the frozen elephant trunk (Figure 3a). Resection of the intimal tear was successful, and remodeling of the descending aorta was observed; however, in 6 months, because re‐entry remained of the continued small true lumen in the aorta at the level of the diaphragmatic junction due to the remaining re‐entry in the distal site of the aorta (Figure 3b,c), TEVAR was performed (Figure 3d). He is currently doing well without any symptoms at the 5‐year follow‐up.

**FIGURE 3:**
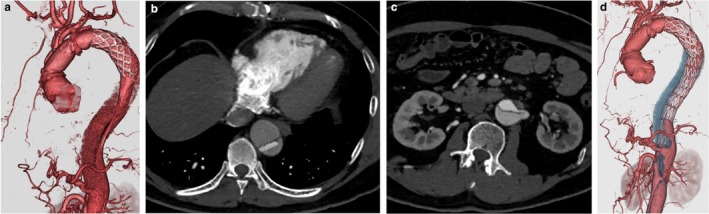
(a) Postoperative computed tomography showing resection of intimal tear, while it shows a narrow true lumen in the aorta at the level of the diaphragmatic junction. (b) Postoperative computed tomography showing remained narrow true lumen in the aorta at the level of the diaphragmatic junction. (c) Postoperative computed tomography showing reentry in the abdominal aorta. (d) Post thoracic endovascular aortic repair computed tomography showing improvement of the narrow true lumen in the aorta at the level of the diaphragmatic junction.

## 3 Discussion

Spontaneous RTAAD has been reported in 7%–25% of ATAAD [3]. According to Kaji et al., RTAAD seems to have a better prognosis than the antegrade form [3, 5]. This may be explained by the less frequent involvement of the aortic valve, coronary arteries, and supra‐aortic trunks [3]. Conversely, Lopez et al. reported that RTAAD was often associated with more extensive distal involvement and higher presentation with malperfusion due to the presence of intimal tears in the distal aorta [3].

Yammine et al. reported that aortic diameter of 40 mm or more is a common risk factor for RTAAD [8]. Williams et al. demonstrated a correlation between ascending aortic diameter and retrograde dissection [9]. Furthermore, morphological abnormalities of the aortic root, such as the obliteration of the sinotubular junction, have been reported to be associated with RTAAD [10]. Moreover, RTAAD occurs more frequently in patients with aortic dissection than in those with true aortic aneurysms, and more frequently in patients with acute dissection than in those with chronic aortic dissection in TEVAR [11].

Patients transported to hospitals are exposed to physiological stressors that may exacerbate hemodynamic instability or induce complications [12]. In the present case, RTAAD developed despite stable vital signs and the absence of symptoms during transfer. Sudden‐onset severe chest or back pain remains the most common symptom of aortic dissection [4]. However, some authors reported that 5%–15% of patients with AAD do not report typical pain [13, 14]. It has been reported that some cases of RTAAD do not present with the typical chest and back pain associated with acute aortic dissection at onset [15]. Ruan et al. reported that RTAAD may be misdiagnosed due to its atypical symptoms, which could lead to catastrophic outcomes [4]. In the present case, the ascending aorta measured 42 mm in diameter and exhibited ATBAD; this suggested a risk of RTAAD. Whether routine CT scanning should be performed after transfer remains controversial. Although post‐arrival CT scanning is not mandatory, additional evaluation should be considered when the above risk factors are present.

The optimal treatment for RTAAD is not standardized with a small series of different approaches available in the literature [5, 6, 7, 16, 17]. Patients with RTAAD accompanied by a thrombosed false lumen in the ascending aorta can be treated with medical therapy followed by scheduled surgical repair [5]. TEVAR may be a useful alternative surgical option in patients with entry into the descending aorta [18]. Kato et al. reported successful endovascular treatment using stent grafts in 10 patients with RTAAD in whom the false lumen in the ascending and descending aorta had completely thrombosed at 3 months after stent grafting [19]. However, these approaches require optimal anatomy, and serious late complications, such as re‐dissection in the ascending aorta, remain a major concern [18]. Therefore, Omura et al. recommended total arch replacement using the fresh or frozen elephant trunk technique for RTAAD in patients without extremely high surgical risks [18].

The standard approach for ATAAD is surgical replacement with complete tear excision; however, in most RTAAD cases where the primary tear is located in the distal descending aorta, it is difficult to achieve this objective via a median sternotomy [7]. Therefore, it is anticipated that ascending aortic replacement alone will not be sufficient to complete the entry closure. Compared with the fresh ET procedure, FET is thought to be a rational option for patients with RTAAD because it reliably covers the primary tear and allows for expansion of the true lumen [7]. Tamura et al. reported that TARFET for RTAAD yielded acceptable outcomes, despite the high preoperative morbidity [6]. TARFET should be selected when considering factors such as intraoperative hemostasis at the anastomosis site and the achievement of postoperative remodeling. However, when using the FET procedure, spinal cord injury is one of the most critical issues that must be considered, and FET insertion between the T6 and T8 levels is recommended to avoid the risk of spinal cord injury [7, 18, 20, 21]. Consequently, covering the primary tear cannot be completed in patients with RTAAD with a tear beneath the level of T9 using this approach [7]. However, the authors propose that even if the primary tear is left open in such patients, the true lumen extending from the distal arch to the proximal descending aorta will likely expand because of its radial self‐expansion force, which may limit the late aortic concerns to more distal sites of the descending aorta [7]. Based on the above discussion, we chose TARFET to complete the entry resection. In the present case, although complete entry resection was possible during the initial surgery, a minor re‐entry remained, necessitating TEVAR for the shrinkage in the aorta at the level of the diaphragmatic junction. Thus, residual stenosis of the true lumen due to a small re‐entry can occur with any surgical technique. In clinical practice, the selection of an appropriate surgical approach for RTAAD is a matter of debate and should be determined based on the patient's condition [7].

Lopez‐Marco et al. state that thorough analysis of CT images, multidisciplinary review in cases of uncertainty, and intraoperative visualization of the entry tear are key to surgical success [3]. They have recently adopted the use of a single‐use flexible bronchoscope during the circulatory arrest to identify the presence of entry and re‐entry tears [3]. They believe that this is especially important when there is no primary tear within the proximal aorta or the arch, as it clearly indicates that the dissection has been of retrograde propagation and precludes excluding the primary entry tear within the descending thoracic aorta [3]. In the present case as well, the entry site was evaluated using intraoperative direct echography, which proved effective for assessing the location of entry. One limitation is that the superiority of TARFET cannot be demonstrated in this single case. Further, it is difficult to prove a causal relationship between transfer and the progression of dissection. In any case, the number of patients with RTAAD is limited, and we believe that a multicenter study is necessary to overcome this limitation. However, variations in preoperative evaluation and surgical strategies among institutions may be a contributing factor making the implementation of a multicenter study difficult.

## 4 Conclusion

RTAAD may occur in patients with ATBAD, even if the patient has no symptoms or hemodynamic changes during transfer. TARFET may be effective in treating RTAAD by resecting intimal tears in the distal arch or proximal descending aorta.

## Author Contributions

**Kayo Sugiyama:** conceptualization, data curation, formal analysis, funding acquisition, investigation, methodology, project administration, resources, software, supervision, validation, visualization, writing – original draft. **Hirotaka Watanuki:** data curation, investigation, methodology, writing – review and editing. **Masato Tochii:** writing – review and editing. **Katsuhiko Matsuyama:** writing – review and editing.

## Funding

The authors have nothing to report.

## Disclosure

Some of the phrasing overlaps because I am referencing my own work. I have listed it below: Kayo Sugiyama, Hirotaka Watanuki, Masato Tochii, Yuji Kuge, Daisuke Koiwa, Katsuhiko Matsuyama. Impact of Direct or Indirect Transfer on Acute Type A Aortic Dissection in The Tokai Region of Japan. J Surg Care. 2023;2(1):71–78. https://www.opastpublishers.com/open‐access‐articles/impact‐of‐direct‐or‐indirect‐transfer‐on‐acute‐type‐a‐aortic‐dissection‐in‐the‐tokai‐region‐of‐japan‐6654.html.

## Ethics Statement

The Ethics Committee of Aichi Medical University Hospital approved this case report on February 3rd, 2025.

(Approval Number, #24‐921).

## Consent

Written informed consent was obtained from the patient to publish this report in accordance with the journal's patient consent policy.

## Conflicts of Interest

The authors declare no conflicts of interest.

## Data Availability

The datasets used and/or analyzed in the current study are available from the corresponding author upon reasonable request.
